# Novel Neuroactive Steroid Analogs and Voltage-Dependent Blockers of Ca_V_3.2 Currents, B372 and YX23, Are Effective Anti-Nociceptives with Diminished Sedative Properties in Intact Female Mice

**DOI:** 10.3390/biom15081175

**Published:** 2025-08-16

**Authors:** Benjamin Volvovitz, Rakib Miah, Kibeom Park, Jae Hun Kim, Raul Vargas, Yuanjiang Xu, Mingxing Qian, Douglas F. Covey, Slobodan M. Todorovic, Vesna Jevtovic-Todorovic

**Affiliations:** 1 Department of Anesthesiology, University of Colorado Anschutz Medical Campus, Aurora, CO 80045, USA; mdrakib.miah@cuanschutz.edu (R.M.); slobodan.todorovic@cuanschutz.edu (S.M.T.); 2 Department of Anesthesiology and Pain Medicine, Keimyung University Dongsan Hospital, School of Medicine, Keimyung University, 1035, Dalgubeol-daero, Dalseo-gu, Daegu 42601, Republic of Korea; parkkibum@dsmc.or.kr; 3 Department of Anesthesiology and Pain Medicine, Konkuk University Medical Center, Konkuk University School of Medicine, 120-1, Neungdong-ro (Hwayang-dong), Gwangjin-gu, Seoul 05030, Republic of Korea; painfree@kuh.ac.kr; 4 Department of Pharmacology, University of Colorado Anschutz Medical Campus, Aurora, CO 80045, USA; raul.vargas@cuanschutz.edu; 5 Department of Developmental Biology, School of Medicine, Washington University in St. Louis, St. Louis, MO 63130, USA; yuanjian@wustl.edu (Y.X.); mqian@wustl.edu (M.Q.); dcovey@wustl.edu (D.F.C.); 6 Taylor Family Institute for Innovative Psychiatric Research, School of Medicine, Washington University in St. Louis, St. Louis, MO 63130, USA; 7 Neuroscience Graduate Program, University of Colorado Anschutz Medical Campus, Aurora, CO 80045, USA; 8 Rocky Mountain Regional VA Medical Center, Aurora, CO 80045, USA

**Keywords:** pain, calcium channels, sedation/hypnosis, mechanical sensitivity, GABA

## Abstract

Although opioids are effective in treating pain, they cause serious side effects. The use of regional anesthesia, although effective in the perioperative period, may not be suitable if mobility and lack of numbness is desired. Hence, there is a clear need for novel pain therapies. Low-voltage activated (T-type) calcium channels (Ca_V_3.2 isoform) could be a promising therapeutic target for the development of novel pain therapies. Indeed, our published findings suggest that novel neuroactive steroid (NAS) analogs that modulate the activity of Ca_V_3.2 channels have unique anti-nociceptive properties. However, the concern with current NASs appears to be their hypnotic/sedative properties, thus potentially hindering the future development of NASs for novel pain therapies. Hence, we developed a new line of NASs that are effective blockers of neuronal Ca_V_3.2 channels in pain pathways while having more favorable pharmacodynamic properties, i.e., lack of sedative/hypnotic side effects. We present two promising novel analogs of NASs—B372 ((3β,5α,17β)-3-Hydroxyandrostan-17-carbonitrile) and YX23 ((3β,5α,17β)-3-Methoxyestran-17-ol). Using an in vitro approach, we show that B372 and YX23 are effective in blocking Ca_V_3.2 channels. Using an in vivo approach, we show that they are effective anti-nociceptives in wild-type but not Ca_V_3.2 knock-out mice. Importantly, we show that they lack sedative/hypnotic effects.

## 1. Introduction

The use of opioids in the operating rooms and outpatient clinics has steadily increased over the past decades making them one of the most commonly prescribed classes of drugs in the USA [1]. Commonly used injectable general anesthetics do not provide a level of analgesia that is required for surgical procedures, necessitating the use of other agents such as opioid analgesics in the perioperative period. Although opioids are very effective in treating the acute pain, they are only partially effective for more chronic use, and their use is associated with side effects including constipation, urinary retention, impaired cognitive function, respiratory depression, tolerance and addiction [2]. Often, even a relatively brief use of opioids in the perioperative period could result in tolerance and drug-seeking behavior. It is estimated that more than 12 million people in the United States abused opioids as of 2010 resulting in more overdose deaths than heroin and cocaine combined [2,3].

Other currently available medications have either limited efficacy or serious side effects. For example, regional anesthesia and use of local anesthetics may not be suitable if early mobility after surgery is desirable. Specifically, prolonged numbness and impaired mobility may prevent early neurological assessment after surgery [4]. Thus, further research into new therapeutic modalities for the treatment of pain is warranted.

The important role of voltage-gated calcium channels (VGCCs) in pain processing has been known for a while since calcium (Ca^2+^) is the major trigger for the release of synaptic vesicles from neuronal presynaptic terminals in response to noxious stimulation [5]. An increase in intracellular Ca^2+^ in pain sensing neurons (nociceptors) can also influence the excitability of these cells [6]. Our studies have shown that the blockade of Ca_V_3.2 isoform of T-type VGCCs in nociceptive dorsal root ganglion (DRG) neurons could be an important cellular target for anti-nociceptive action [7].

Of particular interest for this study is our previously published finding that a class of 5β-reduced NASs can modulate the activity of the Ca_V_3.2 isoform of T-type VGCCs which is considered to underly their potent anti-nociceptive effects in animals with paw skin incision [8]. Specifically, we have shown that NAS, 3β-OH [(3β,5β,17β)-3-hydroxyandrostane-17-carbonitrile] in addition to its hypnotic properties, also displays excellent analgesia in a clinically relevant rodent model of skin incision when administered intrathecally, systemically or peripherally [8]. In addition, we have shown that NASs that inhibit Ca_V_3.2 channels such as ECN [(3β,5α,17β)-17-hydroxyestrane-3-carbonitrile] are effective in alleviating mechanical hyperalgesia post-surgery when administered intrathecally and preemptively whereas morphine provides dose-dependent pain relief only when administered once the pain had developed [9]. Thus, 3β-OH and related NASs such as ECN may represent a novel class of compounds having desirable and unique anti-nociceptive properties following local, systemic or intrathecal delivery.

Having said that, the concern regarding the usefulness of current NASs in the management of nociception appears to be two-fold: (1) their limited aqueous solubility and, (2) the potent hypnotic/sedative properties linked to their effect on gamma aminobutyric acid (GABA)_A_ receptors thus potentially hindering future development of NASs for novel pain therapies in an outpatient setting. For example, although 3β-OH lacks any direct effect on synaptic and extra-synaptic GABA_A_ receptors, we have shown that a sex-specific hypnotic effect of 3β-OH is largely mediated by its peripheral metabolism into an active metabolite, (3α,5β,17β)-3-hydroxyandrostane-17-carbonitrile (3α-OH) that is a potent positive allosteric modulator of neuronal GABA_A_ receptors [10].

Hence, the impetus for this study was to develop novel analogs of NASs that block neuronal Ca_V_3.2 in pain pathways (which underlies effective analgesia) while having more favorable pharmacokinetic (better aqueous solubility) and pharmacodynamic properties (lack of sedative/hypnotic side effects), thus being more usable in outpatient and perioperative clinical settings. We identified two promising novel analogs of NASs: **B372**** ((3β,5α,17β)-3-Hydroxyandrostan-17-carbonitrile**), a 5α-steroid which is the 5α epimer of 3β-OH and, **YX23**** ((3β,5α,17β)-3-Methoxyestran-17-ol)**, an analog of (+)-ECN. Here we examined their blocking effects on Ca_V_3.2 channels in vitro and their anti-nociceptive effects and hypnotic properties in vivo.

## 2. Materials and Methods

### 2.1. Animals

Adult female C57BL/6J mice were purchased from Jackson Laboratories (Bar Harbor, MR, USA). Ca_V_3.2 knock-out (KO) mice were purchased from Jackson Laboratories and were then bred in-house. All animals—wild-type (WT) and KO mice—were housed in a 14:10 light–dark cycle and given access to food and water ad libitum. All experiments were approved by the Institutional Animal Care and Use Committee (IACUC) at the University of Colorado Anschutz Medical Campus and adhered to the National Institutes of Health (NIH) Guide for the Care and Use of Laboratory Animals [11].

### 2.2. Drug Preparation

#### 2.2.1. Synthesis of YX23 (3β,5α,17β)-3-Methoxyestran-17-ol


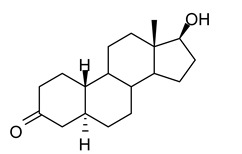


**(5α,17β)-17-Hydroxyestran-3-one (2).** Small pieces of lithium (630 mg) were added to a three-neck round bottom flask. The flask was equipped with a jacketed condenser containing a dry ice/acetone coolant. Anhydrous NH_3_ was condensed into the flask to obtain liquid NH_3_ (~150 mL) at −78 °C. THF (50 mL) was added, followed by the 17β-hydroxyestr-4-en-3one (1.5 g, 18.2 mmol) dissolved in THF and added in three portions (50 mL + 10 mL + 10 mL). After 1 h, solid NH_4_Cl (10 g) was added, the reaction was allowed to warm to room temperature (ca. 23 °C) and the ammonia allowed to evaporate overnight. Water was added to the flask contents, and the product was extracted into EtOAc (200 mL × 2). The combined extracts were dried over anhydrous Na_2_SO_4_ and filtered. The solvent was removed on a rotary evaporator under reduced pressure and the residue was purified by flash column chromatography (silica gel, eluted with 20% EtOAc in hexanes) to give product **2** (3.47 g, 69%): ^1^H NMR (400 MHz, CDCl_3_) δ 3.65–3.61 (m, 1H), 0.75 (s, 3H), 2.40–0.67 (m, 24H); ^13^C NMR (100 MHz, CDCl_3_) δ 211.9, 81.8, 49.9, 48.6, 47.7, 45.7, 43.6, 43.0, 41.2, 41.0, 36.5, 33.8, 30.5, 30.4, 30.1, 25.7, 23.1, 11.0.


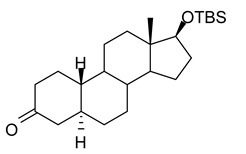


**(5α,17β)-17-((*****tert*****-Butyldimethylsilyl)oxy)-estran-3-one (3).** To a solution of steroid **2** (1.57 g, 5.7 mmol) in DMF (20 mL) was added TBSCl (1.29 g, 8.5 mmol) and imidazole (0.77 g, 11.4 mmol) at room temperature (~23 °C). After 16 h, saturated aqueous NaHCO_3_ (ca. 200 mL) was added and the product was extracted into EtOAc (100 mL). The EtOAc was washed with brine (100 mL × 3), dried over anhydrous Na_2_SO_4_ and filtered. The solvent was removed on a rotary evaporator under reduced pressure and the residue was purified by flash column chromatography (silica gel, eluted with 10% EtOAc in hexanes) to give product **3** (2.10 g, 94%): ^1^H NMR (400 MHz, CDCl_3_) δ 3.56–3.52 (m, 1H), 2.36–0.65 (m, 23H), 0.86 (s, 9H), 0.72 (s, 3H), 0.00(s, 3H), −0.01 (s, 3H); ^13^C NMR (100 MHz, CDCl_3_) δ 211.9, 81.7, 49.5, 48.6, 47.9, 45.7, 43.7, 43.3, 41.3, 41.0, 36.9, 33.9, 30.8, 30.5, 30.2, 25.8 (3C), 25.8, 23.3, 18.0, 11.3, −4.5, −4.9.


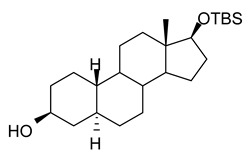


**(3β,5α,17β)-17-((*****tert*****-Butyldimethylsilyl)oxy)-estran-3-ol (4).** To a solution of steroid **3** (2.1 g, 5.4 mmol) in THF (50 mL) was added Lithium tri-*tert*-butoxyaluminum hydride (7 mL, 1.0 M in THF, 7 mmol) at −40 °C. After 1.5 h, water (ca. 200 mL) was added and the reaction was allowed to rise to room temperature (~23 °C). After 16 h, the product was extracted into dichloromethane (100 mL × 3). The combined extracts were washed with water (100 mL), dried over anhydrous Na_2_SO_4_ and filtered. The solvent was removed on a rotary evaporator under reduced pressure and the residue was purified by flash column chromatography (silica gel, eluted with 20% EtOAc in hexanes) to give product **4** (1.90 g, 90%): ^1^H NMR (400 MHz, CDCl_3_) δ 3.56–3.51 (m, 1H), 2.36–0.65 (m, 25H), 0.87 (s, 9H), 0.70 (s, 3H), 0.00 (s, 3H), −0.01 (s, 3H); ^13^C NMR (100 MHz, CDCl_3_) δ 81.8, 70.5, 49.8, 48.2, 46.2, 43.4, 43.3, 41.3, 41.2, 37.1, 35.7, 33.5, 30.9, 30.6, 28.4, 25.8 (3C), 25.6, 23.3, 18.1, 11.3, −4.5, −4.9.


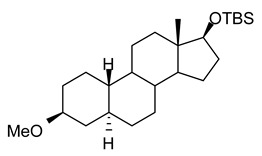


**(3β,5α,17β)-3-Methoxy-17-((*****tert*****-butyldimethylsilyl)oxy)-estrane (5).** A suspension of sodium hydride (60% in mineral oil, 0.6 g, 15 mmol) and steroid **4** (1.90 g, 4.8 mmol) in THF (100 mL) was refluxed under N_2_ for 1 h. Iodomethane (1.5 mL, 22.5 mmol) was added and the reaction was stirred for 2 h. After cooling to room temperature (~23 °C), water (ca. 200 mL) was slowly added and the product was extracted into EtOAc (100 mL × 2). The combined extracts were dried over anhydrous Na_2_SO_4_ and filtered. The solvent was removed on a rotary evaporator under reduced pressure and the residue was purified by flash column chromatography (silica gel, eluted with 10% EtOAc in hexanes) to give product **5** (1.82 g, 93%): ^1^H NMR (400 MHz, CDCl_3_) δ 3.56–3.52 (m, 1H), 3.34 (s, 3H), 3.13–3.08 (s, 1H), 2.10–0.53 (m, 23H), 0.87 (s, 9H), 0.70 (s, 3H), 0.00 (s, 3H), 0.00 (s, 3H); ^13^C NMR (100 MHz, CDCl_3_) δ 81.9, 79.1, 55.5, 49.8, 48.3, 46.6, 43.4, 41.3, 41.2, 39.6, 37.1, 33.6, 32.1, 30.9, 30.6, 28.4, 25.8 (3C), 25.6, 23.4, 18.1, 11.3, −4.5, −4.8.


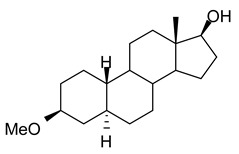


**(3β,5α,17β)-3-Methoxyestran-17-ol** (**6**, **YX23**). To a solution of steroid **5** (1.82 g, 4.47 mmol) in THF (30 mL) was added TBAF (6.7 mL, 1.0 M in THF, 6.7 mmol) at room temperature. The reaction was refluxed for 2 h and the solvent was removed on a rotary evaporator under reduced pressure. The residue was purified by flash column chromatography (silica gel, eluted with 20% EtOAc in hexanes) to give product **6 (YX23)** (1.30 g, 100%): ^1^H NMR (400 MHz, CDCl_3_) δ 3.64–3.59 (m, 1H), 3.33 (s, 3H), 3.13–3.08 (s, 1H), 2.10–0.55 (m, 24H), 0.73 (s, 3H); ^13^C NMR (100 MHz, CDCl_3_) δ 81.9, 79.1, 55.5, 50.1, 48.1, 46.5, 43.0, 41.2, 41.1, 39.5, 36.7, 33.5, 32.1, 30.5, 30.4, 28.4, 25.5, 23.2, 11.0.

#### 2.2.2. Synthesis of B372 (3β,5α,17β)-3-Hydroxyandrostan-17-carbonitrile


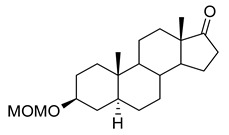


**(3β,5α)-3-(Methoxymethoxy)-androstan-17-one (2).** To a solution of epiandrosterone (**1**, 2 g, 6.9 mmol) in dichloromethane (40 mL) was added chloromethyl methyl ether (13.8 mmol) and (*i*-Pr)_2_NEt (20.7 mmol) at 23 °C. After 16 h, the solvent was removed and the residue was purified by flash column chromatography (silica gel, eluted with 20% EtOAc in hexanes) to give steroid **2** (2.15 g, 93%): ^1^H NMR (400 MHz, CDCl_3_) δ 4.68–4.66 (m, 2H), 3.51–3.45 (m, 1H), 3.35 (s, 3H), 2.46–2.39 (m, 1H), 2.10–2.00 (m, 1H), 1.95–0.90 (m, 20H), 0.84 (s, 3H), 0.82 (s, 3H); ^13^C NMR (100 MHz, CDCl_3_) δ 221.4, 94.5, 76.1, 55.1, 54.4, 51.3, 47.7, 44.8, 36.9, 35.8, 35.7, 35.1, 35.0, 31.5, 30.8, 28.6, 28.4, 21.7, 20.4, 13.7, 12.2.


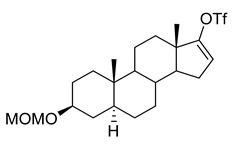


**(3β,5α)-3-(Methoxymethoxy)-androst-16-ene-17-ol,17-(1,1,1 trifluoromethanesulfonate) (3).** To a solution of steroid **2** (2.15 g, 6.4 mmol) in THF (~ 80 mL) was added potassium hexamethyldisilazide (25 mL, 12.5 mmol) and *N*-phenyl bis(trifluoromethanesulfonimide) (5 g, 14 mmol) at −78 °C. After 1 h, the reaction was slowly warmed to 23 °C and after stirring overnight, the reaction was quenched with water (ca. 200 mL) and brine. The product was extracted into EtOAc (150 mL × 3). The solvent was removed, and the residue was purified by flash column chromatography (silica gel, eluted with 10% EtOAc in hexanes) to give steroid **3** (2.57 g, 86%): ^1^H NMR (400 MHz, CDCl_3_) δ 5.53 (s, 1H), 4.65 (s, 2H), 3.50–3.45 (m, 1H), 3.34 (s, 3H), 2.20–2.16 (m, 1H), 1.98–0.72 (m, 19H), 0.93 (s, 3H), 0.82 (s, 3H); ^13^C NMR (100 MHz, CDCl_3_) δ 159.3, 121.0 (q, *J* = 291.4 Hz), 114.4, 94.5, 76.0, 55.0, 54.7, 54.2, 45.0, 44.8, 36.7, 35.8, 35.1, 33.4, 32.6, 30.8, 28.5, 28.5, 28.4, 20.4, 15.2, 12.1.


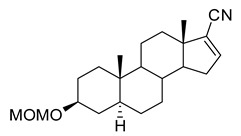


**(3β,5α)-3-(Methoxymethoxy)-androst-16-ene-17-carbonitrile (4).** To a solution of steroid **3** (2.57 g, 5.5 mmol) was added Cu (I) I (100 mg), NaCN (940 mg) and tetrakis(triphenylphosphine)palladium (260 mg) under a nitrogen atmosphere. The reaction was refluxed for 2 h and cooled to room temperature. Aqueous saturated NaHCO_3_ (40 mL) and water (100 mL) were added and the product was extracted into EtOAc (100 mL × 3). The combined organic layers were washed with brine (100 mL), dried over anhydrous Na2SO4, filtered and the solvent was removed. The residue was purified by flash column chromatography (silica gel, eluted with 10% EtOAc in hexanes) to give steroid **4** (1.65 g, 87%): ^1^H NMR (400 MHz, CDCl_3_) δ 6.6 (s, 1H), 4.67 (s, 2H), 3.51–3.46 (m, 1H), 3.36 (s, 3H), 2.36–2.29 (m, 1H), 2.11–2.04 (m, 1H), 1.91–0.72 (m, 18H), 0.90 (s, 3H), 0.84 (s, 3H); ^13^C NMR (100 MHz, CDCl_3_) δ 147.4, 127.4, 115.9, 94.5, 76.0, 55.8, 55.1, 54.6, 48.2, 44.9, 36.7, 35.8, 35.1, 34.0, 33.9, 32.8, 31.7, 28.5, 28.4, 20.8, 16.3, 12.1.


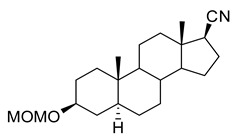


**(3β,5α,17β)-3-(Methoxymethoxy)-androstan-17-carbonitrile (5).** To a solution of steroid **4** (1.65 g, 4.8 mmol) in EtOAc (150 mL) was added Pd/C (10%, 200 mg) in a Parr hydrogenation flask; hydrogenation was continued at 55 psi H_2_ overnight. The mixture was filtered through Celite and washed with EtOAc. The solvent was removed and the residue was purified by flash column chromatography (silica gel, eluted with 20–50% EtOAc in hexanes) to give steroid **5** (1.63 g, 98%): ^1^H NMR (400 MHz, CDCl_3_) δ 4.67 (s, 2H), 3.51–3.46 (m, 1H), 3.36 (s, 3H), 2.28–2.24 (m, 1H), 2.11–2.08 (m, 1H), 1.96–0.65 (m, 21H), 0.90 (s, 3H), 0.82 (s, 3H); ^13^C NMR (100 MHz, CDCl_3_) δ 121.4, 94.5, 76.1, 55.1, 54.3, 54.0, 44.7, 44.4, 40.2, 37.1, 36.9, 35.8, 35.6, 35.1, 31.9, 28.6, 28.5, 26.5, 24.5, 20.8, 14.3, 12.2.


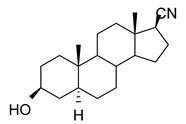


**(3β,5α,17β)-3-Hydroxyandrostan-17-carbonitrile (6, B372).** To a solution of the steroid **5** (1.63 g, 4.7 mmol) in methanol (40 mL) was added acetyl chloride (2 mL) at room temperature. After 2 h, water (20 mL) was added and the product was extracted into dichloromethane (100 mL × 2). The combined organic layers were washed with brine (50 mL × 3), dried over anhydrous Na_2_SO_4_ and the solvent removed. The residue was purified by flash column chromatography (silica gel, eluted with 20% EtOAc in hexanes) to give **B372** (1.31 g, 93%): ^1^H NMR (400 MHz, CDCl_3_) δ 3.56–3.54 (m, 1H), 2.26–1.90 (m, 1H), 1.79–0.61 (m, 23H), 0.88 (s, 3H), 0.79 (s, 3H); ^13^C NMR (100 MHz, CDCl_3_) δ 121.3, 70.9, 54.2, 53.9, 44.6, 44.3, 40.1, 37.9, 37.0, 36.8, 35.7, 35.4, 31.8, 31.2, 28.3, 26.4, 24.4, 20.8, 14.2, 12.2.

All NASs used for our in vivo study were dissolved in 15% (2-hydroxypropyl)-β-cyclodextrin solution (Santa Cruz Biotechnology Inc., Dallas, TX, USA). Drugs were delivered either via intraperitoneal injection (i.p.) at a volume of 10 µL/g or intraplantar (i.pl) injection at a volume of 20 µL.

### 2.3. Behavioral Tests

*a.* 
*Electronic Von Frey*

Mechanical sensitivity was assessed using an electronic Von Frey apparatus (Ugo Basile, Stoelting, Wood Dale, IL, USA). The withdrawal threshold was defined as the minimum force (in grams) required to elicit a clear withdrawal response. Measurements were taken in a standardized sequence, left (injected) hind paw then right (non-injected) hind paw. The testing took place over two days.

*Day 1: Baseline Assessment*

**Figure 1:**
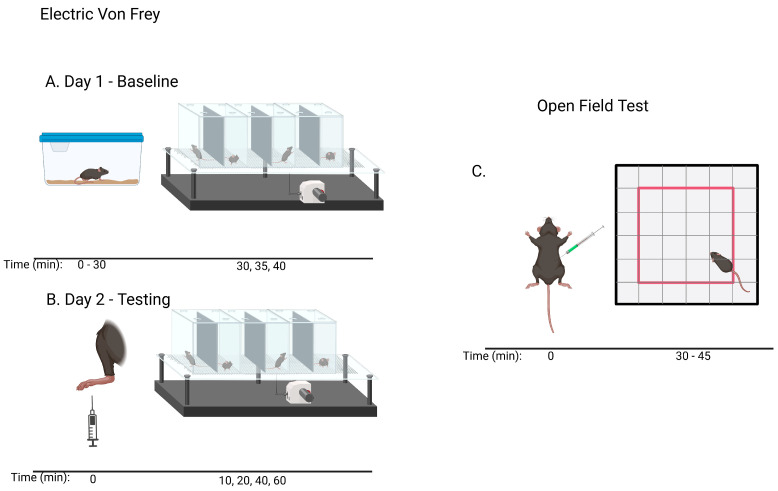
*Schematic representation of the in vivo testing protocols.* (**A**) Baseline assessment of the mechanical sensitivity using an electronic Von Frey apparatus. (**B**) Post-drug injection assessment of the mechanical sensitivity using an electronic Von Frey apparatus up to 60 min post-drug injection into the plantar surface of the hind paw of intact adult female mice. (**C**) Open Field Test used ANY-maze video tracking software to determine the time spent in the outer and the inner zone. Total distance traveled and the velocity were also tracked by ANY-maze software (version 7.5). The observation lasted 15 min post-intraperitoneal drug injection in intact adult female mice. Created in BioRender. Volvovitz, B. (2025) https://BioRender.com/hyfynir. (accessed on 30 June 2025).

Mice were habituated to the testing room in their home cages for 30 min prior to testing. Animals were then placed individually in clear plexiglass chambers on an elevated mesh platform allowing access to the plantar surface of the hind paws. After a 30 min acclimation period to the testing apparatus, mechanical sensitivity was assessed by applying the electronic von Frey filament to the mid-plantar region of each hind paw and the measurements were taken at three time points thereafter (30, 35, and 40 min), with one application per hind paw at each time point. The schematic diagram of the testing protocol is presented in Figure 1A.

*Day 2: Post-injection Assessment*

The baseline assessment protocol from Day 1 was repeated, followed by a 2 h rest period in their home cages. Von Frey testing begins with mice receiving a 20 µL i.pl. hind paw injection of either NAS (10 µM YX23 or 100 µM B372) or a vehicle ((2-hydroxypropyl)-β-cyclodextrin, 15% solution). Immediately after injection, mice were placed in the testing apparatus. Mechanical sensitivity was assessed in both the injected and contralateral hind paws at 10, 20, 40, and 60 min post-injection, with three applications per hind paw at each time point. The experimenter was blinded to the treatment but was not blinded to the type of mouse being used or which hind paw had been injected. A schematic diagram of the testing protocol is presented in Figure 1B.

*b.* 
*Open Field Test*

Sedative/hypnotic effects of the NASs were assessed using the open field test. Mice behavior was tracked using ANY-maze video tracking software (Stoelting Co., Wood Dale, IL, USA). The testing apparatus consisted of a square container (40cm × 40cm). Using ANY-maze, the arena was divided into 2 zones. The outer 1/3 was classified as the outer zone and the inner 2/3 was classified as the inner zone. Total distance traveled and average velocity was also tracked by ANY-maze software. The schematic diagram of the testing protocol and apparatus is presented in Figure 1C.

Mice were habituated to the testing room 30 min before the test started. The animals then received either drug (100 mg kg^−1^ YX23 or 60mgkg^−1^ B372) or vehicle ((2-hydroxypropyl)-β-cyclodextrin, 15% solution) via i.p. injection at a volume of 10 μL/g body weight. Thirty minutes post injection, mice were placed within the testing apparatus and allowed to roam freely for 15 min. The arena was thoroughly cleaned with 70% ethanol between subjects to eliminate olfactory cues.

*c.* 
*Loss of Righting Reflex*

Hypnotic effects of NAS were assessed with the Loss of Righting Reflex (LORR). Animals received drug via i.p. injection at a volume of 10 μL/g body weight. To assess the LORR, mice were placed supine on a flat and level surface 30 min post injection. The assessment window was from 30 to 35 min post injection. The LORR was considered positive if the mouse was unable to return to all four paws on 2 consecutive attempts within 2 min. Only B372 was assessed since neither YX23 nor the vehicle cause the LORR.

### 2.4. Electrophysiology

Human embryonic kidney (HEK-293) cells were stably transfected to express human Ca_V_3.2 channels (Kerafast, Newark, CA, USA). Cells were grown in Dulbecco’s modified Eagle’s medium, 100 U/mL penicillin, and 0.1 mg/mL streptomycin (0.1 mg/mL), and incubated at 37 °C with 5% CO_2_. Media exchange was performed every 2 days, with regular splitting after every 4–7 days (at 85–90% confluency). Prior to recording, cells were split and plated onto poly-D-lysine-coated glass coverslips and allowed to adhere for 2–24 h in an incubator.

For recordings in HEK-293 cells, electrodes were pulled from borosilicate microcapillary tubes to a final resistance of 3–5 MΩ. To record T-currents, electrodes were filled with internal solution comprising 135 mM tetramethylammonium hydroxide, 10 mM ethylene glycol tetraacetic acid (EGTA), 2 mM MgCl_2_, and 40 mM N-2-hydroxyethylpiperazine-N′-2-ethanesulfonic acid (HEPES) and titrated in a plastic dish to a pH of 7.15–7.25 using hydrofluoric acid. The HEK-293 culture medium was exchanged with external solution comprising 152 mM tetraethylammonium (TEA) chloride, 10 mM HEPES, and 2 mM CaCl_2_, and titrated to a pH of 7.4 with TEA hydroxide. Gigaohm seals were reached prior to cell opening to obtain the whole-cell configuration. Membrane resistance, access resistance, and cell capacitance were monitored during the formation of the patch and the subsequent recordings. To obtain current-voltage (IV) curves, cells were maintained at a holding potential (V_h_) of −90 mV and subjected to multiple depolarizing steps (V_t_) ranging from −70 mV to +25 mV. The duration of the voltage step was 320 ms. To obtain steady-state inactivation curves, cells were held at V_h_ = −90 mV, hyperpolarized or depolarized (−110 mV to −40 mV) for a duration of 3.5 s, and then brought to a V_t_ = −30 mV.

### 2.5. Data and Statistical Analysis for Patch-Clamp Experiments

Statistical comparisons in our in vitro experiments were made using paired *t*-test. All data are expressed as mean ± standard error of the mean (SEM); *p* values are reported only when statistically significant (<0.05). The percent reductions in peak current at various concentrations of NAS were used to generate concentration-response curves. Mean values were fit to the following Hill-Langmuir function:

(1) PI([Drug]) = PI_max_/(1 + (IC_50_/[Drug])*h*)

where PI_max_ is the maximal percent inhibition of peak current by the drug, IC_50_ is the concentration that produces 50% inhibition, and *h* is the apparent Hill-Langmuir coefficient for inhibition. The fitted values are reported with >95% linear confidence limits.

The voltage-dependence of steady-state inactivation was fit to the Boltzmann equation: I(V) = Imax/(1 + exp[(V − V_50_)/k]), where Imax is the maximal current amplitude, V_50_ is the half-maximal inactivation voltage, and k (units of millivolts) represents the slope factor. Time course of the macroscopic current inactivation (tau) was assessed with a single exponential function of decaying portion of the current waveforms using the equation: f(t) = A1exp(−t/τ1) yielding one time constant (τ1) and its amplitude (A1). If the data showed wide variations in responses, we tested for normality of the dataset by using Kolmogorov–Smirnov test (e.g., Figure 3C).

### 2.6. Data and Statistical Analysis for In Vivo Experiments

Two-tailed independent samples *t*-tests and one-way ANOVA followed by Tukey’s post hoc test were used to compare groups when equal variances were assumed. *α* was set at 0.05, thus *p*-values < 0.05 were considered statistically significant. Data were graphed as mean ± SEM, and level of significance was indicated by elbow connectors with asterisks. GraphPad Prism 9.3 (GraphPad Software Inc., San Diego, CA, USA) was used for all analyses.

## 3. Results

**I.** 
**Novel neuroactive steroid (NAS) analogs**

**Figure 2:**
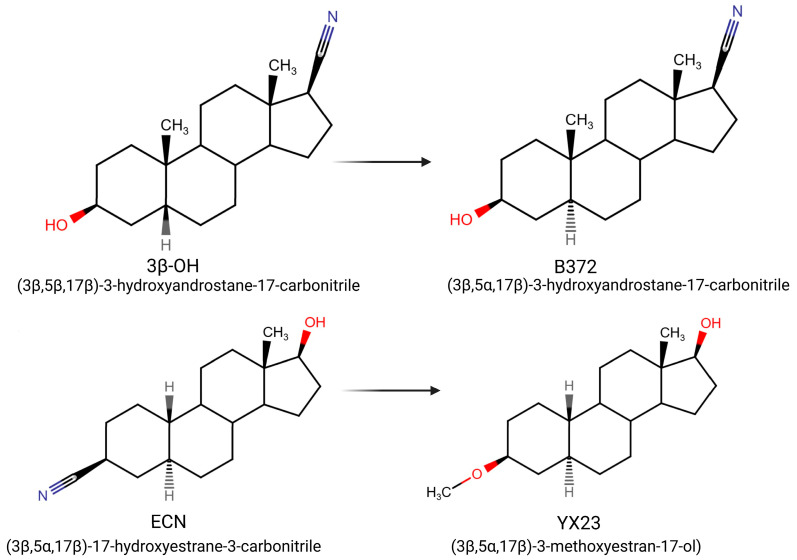
*Chemical structure of parent NASs, 3b-OH and ECN, and their newly synthesized analogs, B372 and YX23, respectively. Created in BioRender. Volvovitz, B. (2025) https://BioRender.com/0sdl6be* (accessed on 30 June 2025).

In this study we focus on two newly synthesized NAS analogs, B372 and YX23 (Figure 2). B372 is the 5α epimer of 3β-OH (a 5β-steroid) (top panels). Although we have shown that 3β-OH is an effective hypnotic [12], efficacious analgesic [7] and a potent voltage-dependent Ca_V_3.2 inhibitor [6], it is in vivo conversion to a potent GABA_A_ positive allosteric modulator, 3α-OH confounds interpretation of how much of the anti-nociceptive effect is due to Ca_V_3.2 inhibition and how much is due to GABA_A_ modulation. To avoid the confounding effect of these dual actions and excessive sedation/hypnosis in vivo we synthesized the new NAS analog, B372, a 5α-steroid and epimer of 3β-OH. In this study we examine the hypnotic and anti-nociceptive properties.

The second NAS analog of interest, YX23 is an analog of ECN (Figure 2, bottom panels), a NAS previously shown to be a potent voltage-dependent Ca_V_3.2 inhibitor [6].

ECN does not have a 3-OH group and unlike 3β-OH cannot be metabolized in vivo to a GABA_A_ modulator. YX23 also does not have a 3β-OH and by replacing the cyano group of ECN with a methoxy group in the 3β position of the steroid ring, YX23 was made a more soluble analog compared to ECN. When we calculated LogP (lipophilicity vs. hydrophilicity) of ECN vs. YX23, we found that LogP value for ECN is **4.04** vs. LogP of **3.59** for YX23 suggesting higher hydrophilicity of YX23 and its higher solubility. In this study we examine its hypnotic and anti-nociceptive properties of B372 and YX23.

**II.**  **Electrophysiology studies** 

**Figure 3:**
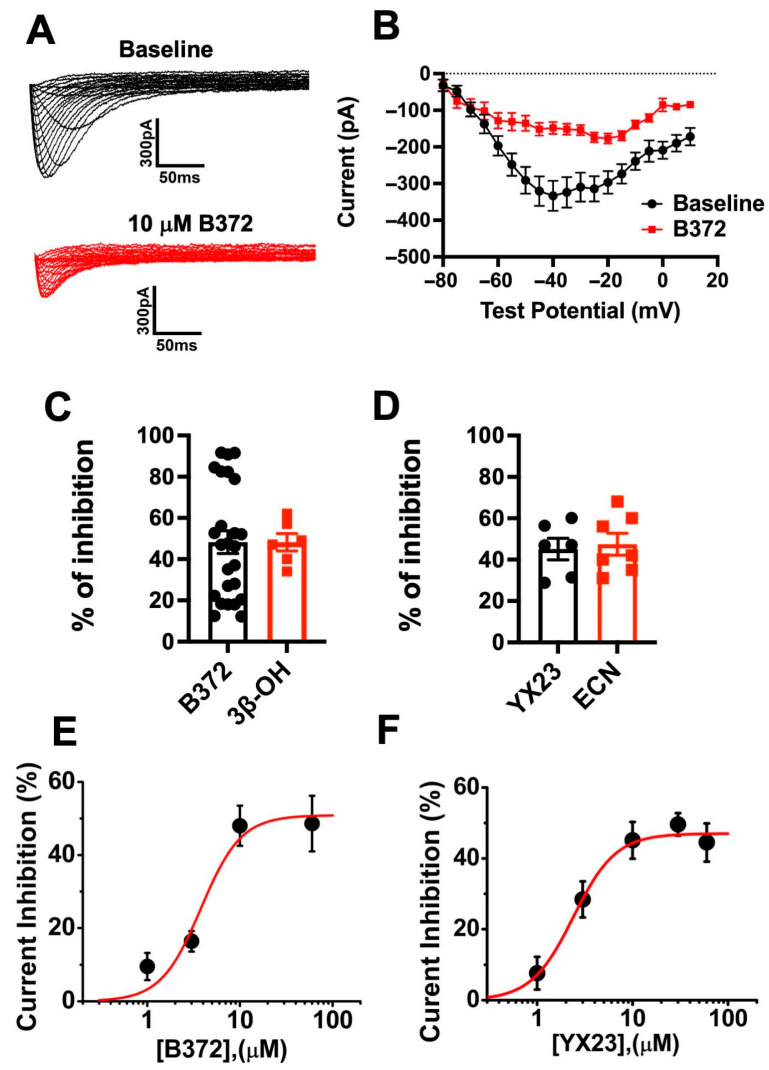
*Concentration-dependent inhibition of recombinant CaV3.2 currents by novel neuroactive steroid analogs.* (**A**) Families of original current traces obtained using our I–V protocols from the same HEK293 cell obtained in our external solution before (black traces) and after application of 10 µM B372 (red traces). Note typical criss-cross patterns caused by faster inactivation of currents at more depolarized test potentials. Insets represent calibration bars. (**B**) Plot shows the average I-V graph from 21 cells using the same protocol depicted on panel (**A**). Averaged date points represent mean ± SEM in baseline pre-drug conditions (black symbols) and following application of 10 µM B372 in external solution. Note that B372 inhibited peak current amplitudes over the wide range of test potentials. (**C**) Bar graphs compare percent of current inhibition by 10 µM B372 (black symbols) and 10 µM 3β-OH (red symbols). Both drugs inhibit on average 48.2 ± 5.5% and 48.2 ± 4.2% of baseline peak currents (*p* > 0.05, two-tailed *t*-test). (**D**) Bar graphs compare percent of current inhibition by 10 µM YX23 (black symbols) and 10 µM ECN (red symbols). Both drugs inhibit, on average, 45.1 ± 5.2% and 47.4 ± 5.2% of baseline peak currents (*p* > 0.05, two-tailed *t*-test). (**E**) Concentration–response curve for the inhibition of Ca_V_3.2 currents by escalating concentrations of B372. Each black symbol is the mean ± SEM of multiple cells (n = 5–26). The solid red line is the best fit of the Hill–Langmuir function, yielding an estimated IC_50_ of 3.8 µM, a slope steepness factor of 2.0, and a maximal current inhibition of 50.9%. (**F**) Concentration-response curve for the inhibition of Ca_V_3.2 currents by escalating concentrations of YX23. Each black symbol is the mean ± SEM of multiple cells (n = 5–8). Solid red line is the best fit of Hill–Langumir function yielding an estimated IC_50_ of 2.4 µM, slope steepness factor of 1.9 and maximal current inhibition of 47.0%.

To examine the hypothesis that B372 and YX23 are Ca_V_3.2 inhibitors, we performed patch-clamp studies using stably transfected HEK-293 cells. For screening purposes, these NASs were dissolved in 100% dimethyl sulfoxide (DMSO) as 10–20 mM stock solutions; for actual recordings, they were freshly diluted in our external solution to final concentrations ranging from 1 to 60 µM. Maximal concentration of DMSO in external solutions used for recordings was 0.3%, which we previously demonstrated had minimal effect on Ca_V_3.2 currents in HEK 293 cells [13]. Our current-voltage (I–V) protocol for recordings of recombinant Ca_V_3.2 currents is depicted in Figure 3A, which shows two families of inward currents. Black traces in Figure 3A show baseline pre-drug currents, and red traces show currents in the same cell after application of 10 µM B372. The peak inward currents from this representative experiment were plotted on average on the I-V curve from similar experiments depicted on Figure 3B. Note that B372 inhibited about 50% of peak current amplitudes over the range of potentials with maximal inhibition occurring between Vt −40 and Vt −20 mV. Importantly, our patch clamp experiments have established that B372 is an efficacious Ca_V_3.2 inhibitor similar to 3β-OH with both compounds blocking on average about 50% of peak current at 10 µM concentrations (Figure 3C).

Our similar patch-clamp studies with YX23 have shown that YX23, at 10 µM similarly inhibits baseline Ca_V_3.2 currents by about 45% (*p* < 0.001 by two-tailed Student *t*-test, n = 6 cells) (Figure 3D). Importantly, our patch clamp experiments have established that YX23 is an efficacious Ca_V_3.2 inhibitor like ECN with both compounds blocking on average about 45% of peak current at 10 µM concentrations (Figure 3D). From the concentration-response experiments using I–V protocols and escalating concentrations of NASs we estimated maximal block of peak T-currents for each analog, slope of the curve and an IC_50_ for current inhibition. Figure 3E depicts average data points from multiple experiments for B372 which inhibited about 51% of peak T-currents with an IC_50_ of 3.8 µM and slope factor of 2.0. Similarly, Figure 3F depicts average data points from multiple experiments for YX23 which inhibited about 47.0% of peak T-currents with an IC_50_ of 2.4 µM and slope factor of 1.9. Our previous studies demonstrated that both 3β-OH and ECN inhibited T-currents in a voltage-dependent manner [7]. Hence, we evaluated any state-dependent features of Ca_V_3.2 current inhibition by YX23 and B372. Drug binding to inactivated states of ion channels is an important property since it allows for tissue selectivity based on differences in membrane potentials, where more depolarized membranes will have T-channels which cycle through the inactivated state more often vs. less excitable tissue. Transitions from closed to inactivated states can be measured using long prepulses at different potentials, producing what are commonly referred to as steady-state inactivation curves.

**Figure 4:**
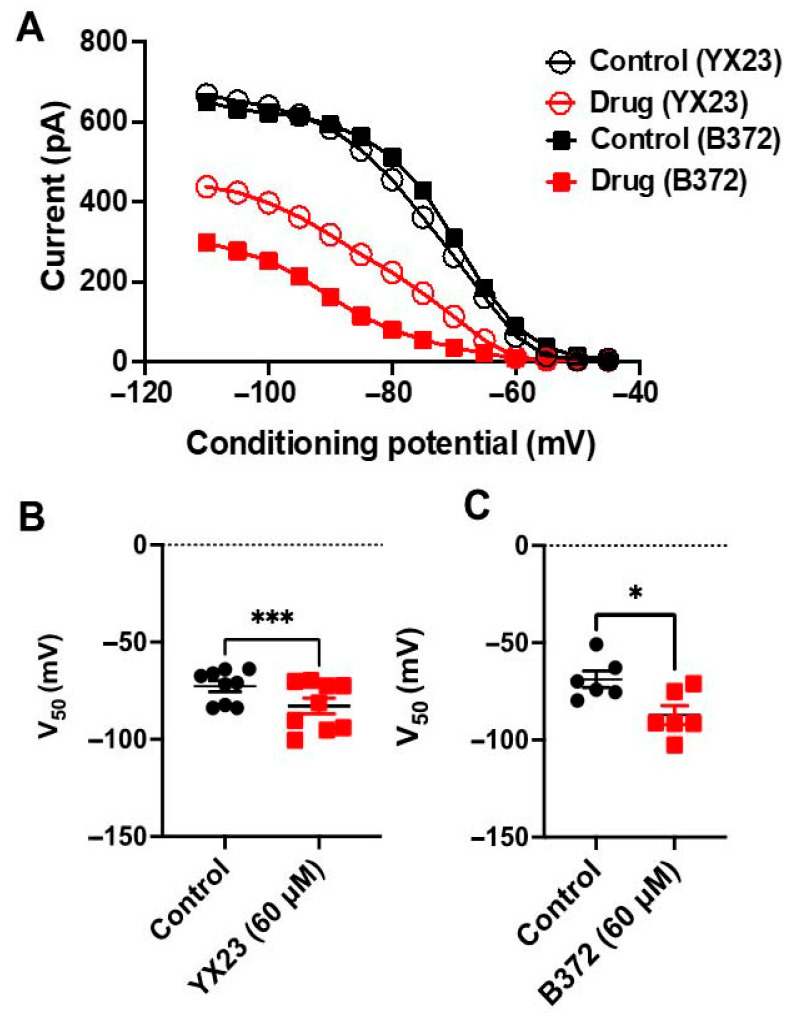
*Voltage-dependent inhibition of recombinant Ca_V_3.2 currents by novel neuroactive steroid analogs.* (**A**) The average Ca_V_3.2 current steady-state inactivation curves from multiple experiments. Black open circles represent the control conditions obtained in our external solution; red open circles represent the conditions after bath applications of 60 µM YX23 in the same cells (n = 9). Black filled squares represent the control conditions; red filled squares represent the conditions after bath applications of 60 µM B372 in the same cells (n = 6). Solid lines are fitted using Boltzmann equation (see Materials and Methods), giving half-maximal availability (V_50_), which occurred at −73.0 mV with a slope *k* of 8.0 mV in control conditions. In contrast, V_50_ was −80.1 mV with a slope *k* of 10.1 mV in the conditions after YX23 was applied. In another set of experiments, we found half-maximal availability (V_50_), which occurred at −70.2 mV with a slope *k* of 6.5 mV in control conditions. In contrast, V_50_ was shifted to more negative potential of −90.0 mV with a slope *k* of 8.8 mV in the conditions after B372 was applied. (**B**) Scatter plot shows V_50_ values in control conditions (black filled circles) and after applications of YX23 (red filled squares) obtained in individual cells by using steady-state inactivation curves as depicted on panel A of this figure. The average V_50_ in the control conditions occurred at −72.7 ± 2.8 mV, while in the presence of YX23, it occurred at −82.9 ± 4.1 mV (***, *p* = 0.0009). (**C**) Scatter plot shows V_50_ values in control conditions (black filled circles) and after applications of B372 (red filled squares), which were obtained in individual cells by using steady-state inactivation curves as depicted on panel A of this figure. The average V_50_ in the control conditions occurred at −68.8 ± 4.2 mV, while in the presence of B372, it occurred at −87 ± 4.8 mV (*, *p* = 0.028).

Figure 4A shows that when compared to control predrug conditions both NASs decreased T-current amplitudes over all tested preconditioning potentials. Furthermore, when compared to control predrug conditions, both YX23 and B372 had a great effect on the voltage-dependent kinetics of channel inactivation, as determined by a hyperpolarizing shift in steady-state inactivation curves of about 10 mV as shown on Figure 4B and Figure 4C, respectively. These data suggest that B372 and YX23 bind to and stabilize inactive states of the Ca_V_3.2 T-channel and thus are more potent blockers at depolarized membrane potentials. For example, in 4A we show that 60 µM YX23 inhibits only about 30% of maximal T-current at −110 mV, while the same concentration inhibits about 90% T-current at preconditioning potentials of −65 mV. We also compared inactivation time constant (tau) values in control predrug conditions and after application of 60 µM of NAS at preconditioning potentials of –90 mV. We found that both drugs significantly accelerated inactivation as follows: B372 from 31.1 ± 1.0 ms to 23.0 ± 1.9 ms (*p* < 0.01, n = 5, paired-test) and YX23 from 28.7 ± 3.2 ms to 21.1 ± 1.8 ms (*p* < 0.01, n = 7, paired *t*-test).

Based on these data, we concluded that structural modifications of 3β-OH and ECN did not have an apparent adverse impact on their ability to inhibit Ca_V_3.2 current in a voltage dependent manner.

**III.**  **The assessment of novel neuroactive steroid analogs’ sedative/hypnotic properties**

**Figure 5:**
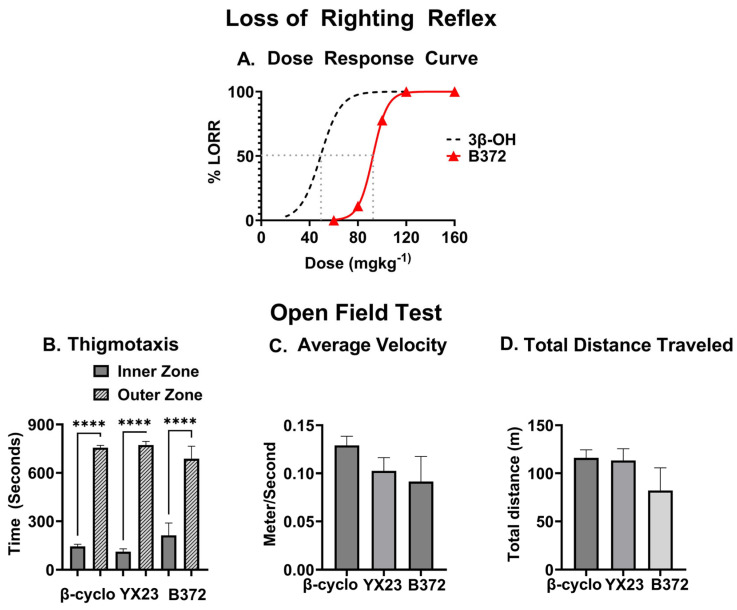
*The assessment of novel neuroactive steroid analogs’ sedative/hypnotic properties.* (**A**) A dose–response experiment with escalating doses of B372 (from 60 to 160 mg/kg given systemically (red line) using LORR in the WT female adult mice. (**B**) Open Field Test using YX23 at 100 mg/kg and B372 at 60 mg/kg, i.p. The time spent in the Inner Zone and Outer Zone (Thigmotaxis) during a 15 min observation post-B372 injection was measured. There was no significant difference compared to vehicle-treated controls (****, *p* < 0.0001). (**C**) Open Field Test using YX23 at 100 mg/kg and B372 at 60 mg/kg, i.p. We found no difference in the average velocity of their movements when compared to the vehicle controls. (**D**) Open Field Test using YX23 at 100 mg/kg and B372 at 60 mg/kg, i.p. We found no difference in the total distance traveled when compared to the vehicle controls.

Our recent study has shown that a systemic injection of 3β-OH produced potent hypnotic effect as measured with loss LORR in the range of doses from 20 to 120 mg/kg, i.p. [10]. Hence, in order to assess the hypnotic potential of its newly synthesized epimer, B372 we performed a dose–response experiment with escalating doses of B372 (from 60 to 160 mg/kg given systemically) (i.p.). We measured LORR in the wild type (WT) female adult mice (n = 9 per group) (Figure 5A). For the easy of comparison, we include as a dotted line, the dose response to 3β-OH that was previously published [10]. We noted a 2-fold rightward shift with B372 (the ED_50_ of 92 mg/kg; i.p.) compared to the published ED_50_ for 3β-OH (48 mg/kg; i.p.), suggesting that the structural modification of the 3β-OH chemical structure greatly diminished the sedative/hypnotic potency of B372.

To further assess systemic effects of B372, we conducted an Open Field Test using B372 at 60 mg/kg, i.p. (injection volume of 10 μL/g of body weight) (n = 9–10 mice per group) (Figure 5B). When the times spent in the Inner Zone and Outer Zone (Thigmotaxis) during a 15 min observation post-B372 injection were measured, we concluded that there was no significant difference compared to vehicle-treated controls (n = 9–10 mice per group). Similarly, we found no difference in the average velocity of their movements (Figure 5C) or total distance traveled (Figure 5D) when compared to the vehicle controls suggesting lack of significant sedative effects or changes in the overall motor abilities of the mice.

Similarly, we found that YX23, at 100 mg/kg, i.p. had no appreciable effect on any of the observed open field behaviors when compared to vehicle-controls (Figure 5B–D). In addition, unlike B372, we did not observe any appreciable signs of sedation or lack of movement when compared to vehicle controls. Hence, we did not perform LORR studies.

**IV.**  **The assessment of novel neuroactive steroid analogs’anti-nociceptive properties in intact animals**

To assess the anti-nociceptive properties of chosen novel NASs, we conducted studies of mechanical sensitivity using an electronic von Frey apparatus applied to the plantar surface of the hind paw in mice (see Methods).

**Figure 6:**
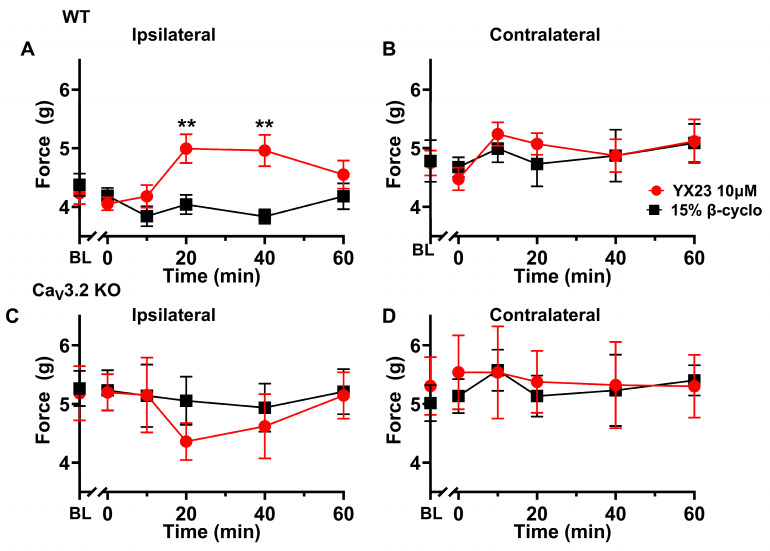
*YX23 is potent peripheral anti-nociceptive agent in intact adult female mice.* (**A**) When a small amount (20 µL) of YX23 (at 10 µM) is injected i.pl. into the receptive field of sensory neurons of an adult WT mouse left paw and PWR to punctate mechanical stimuli as a bending force (in gm) were recorded before injection (0 time point) and at different time points after the injection (10, 20, 40 and 60 min) we noted diminished PWRs as evidenced by significantly increased mechanical thresholds in injected paws at 20 and 40 min when compared to injections of a vehicle (**, *p* < 0.01) (n = 11 female mice per data point). (**B**) Injections of either YX23 or vehicle did not change PWRs in contralateral non-injected paws indicating the lack of a systemic effect (n = 11 female mice per data point). (**C**) When a small amount (20 µL) of YX23 (at 10 µM) is injected i.pl. into the receptive field of sensory neurons of an adult Ca_V_3.2 KO mouse left paw and PWR to punctate mechanical stimuli were recorded before injection (0 time point) and at different time points after the injection (10, 20, 40 and 60 min) we found no significant difference in mechanical sensitivity at any time point after the YX23 injection when compared to vehicle controls (n = 8 female mice per data point). (**D**) Injections of either YX23 or vehicle did not change PWRs in contralateral non-injected paws of adult Ca_V_3.2 KO mouse indicating the lack of a systemic effect (n = 8 female mice per data point) (BL-baseline PWRs).

As shown in Figure 6A we found that the injection of the YX23, at 10 µM showed diminished paw withdrawal response (PWR) as evidenced by significantly increased mechanical thresholds by about 25% in injected paws at 20 and 40 min when compared to injections of a vehicle (**, *p* < 0.01). Importantly, injections of either YX23 or vehicle did not change PWRs in contralateral non-injected paws (Figure 6B) indicating the lack of a systemic effect (n = 11 female mice per data point).

**Figure 7:**
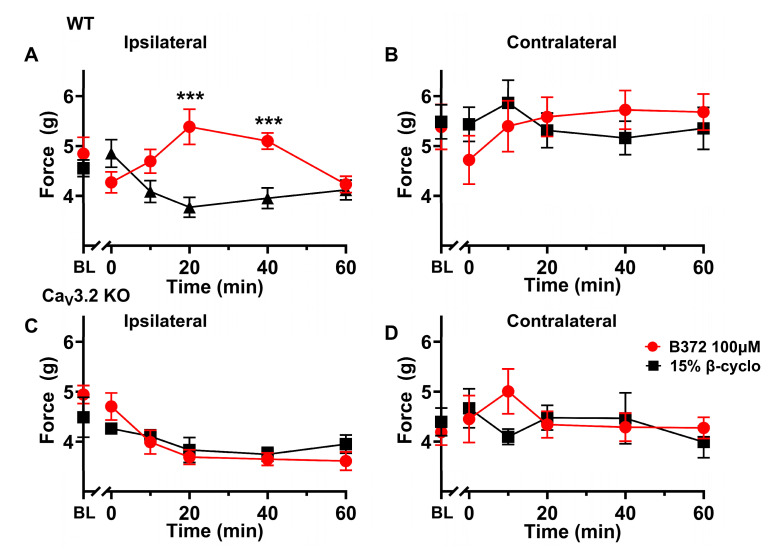
*B372 is potent peripheral anti-nociceptive agent in intact adult female mice.* (**A**) When a small amount (20 µL) of B372 (at 100 µM) is injected i.pl. into the receptive field of sensory neurons of an adult WT mouse left paw and PWR to punctate mechanical stimuli as a bending force (in gm) were recorded before injection (0 time point) and at different time points after the injection (10, 20, 40 and 60 min) we noted diminished PWRs as evidenced by significantly increased mechanical thresholds in injected paws at 20 and 40 min when compared to injections of a vehicle (***, *p* < 0.001) (n = 10 female mice per data point). (**B**) Injections of either B372 or vehicle did not change PWRs in contralateral non-injected paws indicating the lack of a systemic effect (n = 10 female mice per data point). (**C**) When a small amount (20 µL) of B372 (at 100 µM) is injected i.pl. into the receptive field of sensory neurons of an adult Ca_V_3.2 KO mouse left paw and PWR to punctate mechanical stimuli were recorded before injection (0 time point) and at different time points after the injection (10, 20, 40 and 60 min) we found no significant difference in mechanical sensitivity at any time point after the B372 injection when compared to vehicle controls (n = 6 female mice per data point). (**D**) Injections of either B372 or vehicle did not change PWRs in contralateral non-injected paws of adult Ca_V_3.2 KO mouse indicating the lack of a systemic effect (n = 6 female mice per data point) (BL-baseline PWRs).

To further assess the role of Ca_V_3.2 channels in peripheral nociception, we examined the effect of YX23 in Ca_V_3.2 KO mice. As shown in Figure 6C, we found no significant difference in mechanical sensitivity at any time point after the YX23 injection when compared to vehicle controls (n = 8 female mice per data point). The finding was similar in the contralateral paw (Figure 6D), suggesting that YX23’s anti-nociceptive effects are indeed largely mediated by the peripheral Ca_V_3.2 channels. As shown in Figure 7A we found that the injection of the B372, at 100 µM showed diminished PWRs as evidenced by significantly increased mechanical thresholds by about 30% in injected paws at 20 and 40 min when compared to injections of a vehicle (***, *p* < 0.001). Importantly, injections of either B372 or vehicle did not change PWRs in contralateral non-injected paws (Figure 7B) indicating the lack of a systemic effect (n = 10 mice per data point).

To further assess the role of Ca_V_3.2 channels in peripheral nociception we also examined the effect of B372 in Ca_V_3.2 KO mice. As shown in Figure 7C, we found no significant difference in mechanical sensitivity at any time point after the B372 injection when compared to vehicle controls (n = 6 mice per data point). The finding was similar in the contralateral paw (Figure 7D) suggesting that YX23’s anti-nociceptive effects are indeed largely mediated by the peripheral Ca_V_3.2 channels.

## 4. Discussion

In this study we show that two newly synthesized NAS analogs, B372 and YX23 are effective anti-nociceptive agent in intact female animals when injected into the receptive field of sensory neurons of a hind paw at doses that do not cause systemic anti-nociceptive effects. Furthermore, by modifying the chemical structure of their parent NASs, 3β-OH and ECN, respectively, we have designed analogs that when injected systemically, do not cause hypnosis thus making them potentially more useful in the outpatient clinical setting where anti-nociception, without heavy sedation, is a desirable therapeutic goal.

VGCCs serve essential functions in action potential formation, cellular excitability regulation, and synaptic transmission control. These channels are classified based on their activation thresholds: high-voltage-activated channels and low-voltage-activated channels, also known as transient or T-type Ca^2+^ channels [14]. Different genes encode the α1 subunits that constitute the channel pores. The Ca_V_1 family produces L-type channels, while the Ca_V_2 family includes three subtypes: Ca_V_2.1 (P/Q-type), Ca_V_2.2 (N-type), and Ca_V_2.3 (R-type) channels [15]. Research has demonstrated that N-type channel blockers significantly contribute to presynaptic inhibition within the spinal cord’s dorsal horn [16]. Furthermore, knock-out studies using Ca_V_2.1 and Ca_V_2.3 deficient mice have revealed altered pain responses, indicating these channels’ involvement in nociceptive processing [17]. The cloning of T-channel pore-forming α1 subunits has identified three distinct subtypes: Ca_V_3.1 (G-type), Ca_V_3.2 (H-type), and Ca_V_3.3 (I-type) [18]. Among these, Ca_V_3.2 channels have been established as critical mediators of nociception [13,19,20,21], including findings from recent investigations involving plantar skin incision models [9,22,23]. The current research contributes significantly to the existing body of evidence supporting Ca_V_3.2 channels as viable therapeutic targets. The study demonstrates that novel NASs that block Ca_V_3.2 channels provide effective anti-nociception in WT animals while showing no effect in Ca_V_3.2 KO mice demonstrating the importance of Ca_V_3.2 channels in nociceptive pathways.

NASs are potent modulators of neuronal activity by causing a variety of behavioral and neuroendocrine changes in humans and animals (e.g., general anesthesia, analgesia, cognitive and mood disturbances) [24,25]. Interestingly, it is traditionally believed that these effects on neurosensory processing and neuronal excitability are primarily mediated by their potent modulation of GABA_A_ receptors. For example, NAS, alphaxalone ((3α,5α)-3-hydroxypregnane-11,20-dione) which effectively potentiates GABA_A_-gated currents is also a good analgesic [26] but importantly, a potent general anesthetic, a common property of positive allosteric GABA_A_ modulators. However, as previously reported, alphaxalone is also a potent inhibitor of T-currents in dorsal root ganglion (DRG) neurons, suggesting that its analgesic/anti-nociceptive effects could also be due to its blocking effect on T-channels [26]. Although hypnotic effects combined with potent analgesic/anti-nociceptive action could be very beneficial in surgical settings where general anesthesia is desired, in the outpatient clinical setting, the goal of pain alleviation without serious sedation is of paramount importance to assure a good quality of life. Hence, there is a need for the development of effective analgesics/anti-nociceptives that are not only non-addictive but also non-sedative/non-hypnotic.

It is noteworthy that based on our behavioral nociceptive findings reported herein, there is some increase in mechanical sensitivity observed in the hind paw of vehicle-injected animals (when compared to the contralateral, non-injected hind paws). We contribute this observation to be most likely due to the fact that the intraplantar needle injection is uncomfortable (caused by intraplantar skin puncture and subsequent skin stretching with the injectate into the paw) thus making the paw more sensitive to the application of the filament. Since a vehicle, unlike the NAS, lacks anti-nociceptive properties, the reported PWRs appear to be somewhat lower. Having said that, the significant NAS anti-nociceptive properties are evident not only when the ipsilateral sides are compared to the vehicle ones but also based on the findings that NAS injected paw responses to the mechanical stimuli are comparable to the non-injected paws suggesting substantial anti-nociceptive effect of NAS that ‘normalizes’ the responses to the mechanical stimuli despite an uncomfortable injection. Furthermore, since KO animals lack the target channel (Ca_v_3.2), the absence of NAS nociceptive effect results in similar sensitization of the injected paw in both vehicle and NAS-injected animals indicating that Ca_v_3.2 channels play an important role in NAS-induced anti-nociceptive effect.

Of importance for our study presented herein is our previous discovery that a NAS with a 5α configuration at the steroid A,B ring fusion, [(+)-ECN] [(3β,5α,17β)-17-hydroxyestrane-3-carbonitrile] (see structure on Figure 2), is a potent voltage-dependent blocker of Ca_V_3.2 T-channels in rat DRG neurons [27]. Importantly, ECN only weakly inhibits recombinant Ca_V_2.3 currents [28] and has very little effect on voltage gated Na^+^, K^+^, N- and L-type HVA Ca^2+^ channels, glutamate, and GABA-gated channels [27]. Furthermore, we have shown that the analgesic efficacy of alphaxalone, ECN and related 5α-reduced steroids is correlated with their ability to inhibit T-currents in DRG neurons [26]. We have also identified several synthetic 5β-reduced steroid analogs that lack any direct effect on GABA_A_ currents but potently and completely inhibit Ca_V_3.2 currents in DRG cells and exhibit potent analgesic effects in vivo [7]. One of the most potent and efficacious steroid analogs in this group, 3β-OH ((3β,5β,17β)-3-hydroxyandrostane-17-carbonitrile) (Figure 2) is a voltage-dependent and selective blocker of T-currents in acutely dissociated DRG cells [7]. However, limited aqueous solubility (ECN) and potent hypnotic/sedative effects (3β-OH) linked to 3β-OH effects on GABA_A_ receptors may hinder future development of these NASs for novel pain therapies. Specifically, although 3β-OH lacks any direct effect on synaptic and extra-synaptic GABA_A_ receptors, we published that a sex-specific hypnotic effect of 3β-OH after i.p. injections is largely mediated by its peripheral metabolism into an active metabolite, 3α-OH that is a potent positive allosteric modulator of neuronal GABA_A_ receptors [10]. Herein, using two novel NAS analogs, B372 and YX23 with more favorable pharmacokinetic and pharmacodynamic properties, as well as better water solubility, we explore their anti-nociceptive potential as neuronal Ca_V_3.2 inhibitors in pain pathways. We believe that the introduction of novel NAS analogs as presented in this study is an important step toward an intended therapeutic goal, i.e., these novel NAS analogs may ensure that we are a step closer to a promising therapeutic strategy that is non-habit forming and non-sedative.

In this study we focus on female mice since sex differences in pain perception and response to pain are well documented, i.e., females are, in general, more sensitive to a variety of painful conditions thus more commonly requiring the use of an array of analgesics and psychological pain coping strategies [29]. In addition, based on our previous work, sex-specific hypnotic effect of some NASs (i.e., 3β-OH) depends on their peripheral metabolism that seems to be more pronounced in female sex [10] thus resulting in a higher propensity for sedative/hypnotic side effects that could have a negative impact on daily functioning and the quality of life. Hence, we believe that assessing the usefulness of NAS in anti-nociception first in female mice is important while the inclusion of male mice, once equipped with female data, could be beneficial in the future studies. Another limitation of our study is the use of recombinant human Ca_V_3.2 channel for our in vitro studies. Although this method is convenient as it allows studies of well isolated currents, it lacks ability to investigate possible effects of NASs on other nociceptive ion channels in sensory neurons that could contribute to their effects in vivo. Nevertheless, our data relating to mouse genetics indicate that Ca_V_3.2 channels in peripheral nociceptors are required for the anti-nociceptive effects of YX23 and B372 following injections into peripheral receptive fields.

Although not of primary interest for this study, it is noteworthy that previously examined NAS analogs with T-channel blocking properties—3β-OH and ECN—are also recognized as potentially useful drugs for the treatment of chronic pain [30]. When injected systemically or intrathecally, they were reported to ameliorate the development of allodynia, both mechanical and thermal. Further studies with B372 and YX23 in the setting of acute post-surgical and chronic neuropathic pain could be a promising next step, especially since chronic use of opioids is controversial and often contraindicated in this patient population.

## 5. Conclusions

In this study we introduce two newly synthesized NASs, B372 and YX23, which are derivatives of 3β-OH and ECN, respectively. We show that both NASs are reliable Ca_V_3.2 inhibitors in vitro in a recombinant system expressing Ca_V_3.2 channels (HEK293 cells), suggesting that the structural modifications of 3β-OH and ECN do not have an apparent adverse impact on B372’s and YX23’s ability to block Ca_V_3.2 currents in a voltage-dependent fashion. Importantly, we show that B372 and YX23 are good anti-nociceptive agents in intact female mice when administered in the peripheral receptive field of sensory neurons in hind paw skin while lacking systemic anti-nociceptive and/or sedative/hypnotic effects. Considering the serious side effects of currently used opioid drugs, we believe that this study ensures that we are a step closer to the development of effective and non-addictive clinical therapies.

## 6. Patents

U.S. Provisional Application Serial No. 63/695,628 *was filed on 09/17/2024: **STEROID BLOCKERS OF T-TYPE CALCIUM CHANNELS FOR TREATMENT OF PAIN***.

## Data Availability

We will share with the scientific community any compounds upon request following proper material transfer agreement once our results are published and the decisions regarding patent applications are finalized. All raw data will be uploaded to the DANDI archive created by the BRAIN initiative (dandiarchive.org). DANDI is a public archive that allows for the storage and dissemination of neurophysiology and behavioral data once the manuscript is published.

## Abbreviations

The following abbreviations are used in this manuscript:

NAS	Novel neuroactive steroids
B372	(3β,5α,17β)-3-Hydroxyandrostan-17-carbonitrile
BL	Baseline
YX23	(3β,5α,17β)-3-Methoxyestran-17-ol
LORR	Loss of Righting Reflex
Ca^2+^	Calcium
VGCC	Voltage-gated calcium channel
3β-OH	(3β,5β,17β)-3-hydroxyandrostane-17-carbonitrile
ECN	(3β,5α,17β)-17-hydroxyestrane-3-carbonitrile
3α-OH	(3α,5β,17β)-3-hydroxyandrostane-17-carbonitrile
KO	Knock-out
HEK	Human embryonic kidney
PWR	Paw withdrawal response
i.pl.	Intraplantar
i.p.	Intraperitoneal
DMSO	Dimethyl sulfoxide
DRG	Dorsal root ganglion
